# Electro-Active Polymers (EAPs): A Promising Route to Design Bio-Organic/Bioinspired Platforms with on Demand Functionalities

**DOI:** 10.3390/polym8050185

**Published:** 2016-05-09

**Authors:** Vincenzo Guarino, Simona Zuppolini, Anna Borriello, Luigi Ambrosio

**Affiliations:** Institute of Polymers, Composites and Biomaterials, Department of Chemical Sciences and Materials Technologies, National Research Council of Italy, V.le Kennedy 54, 80125 Naples, Italy; simona.zuppolini@unina.it (S.Z.); direttore.dsctm@cnr.it (L.A.)

**Keywords:** conductive polymers, scaffolds, biosensors, molecular release

## Abstract

Through recent discoveries and new knowledge among correlations between molecular biology and materials science, it is a growing interest to design new biomaterials able to interact—*i.e.*, to influence, to guide or to detect—with cells and their surrounding microenvironments, in order to better control biological phenomena. In this context, electro-active polymers (EAPs) are showing great promise as biomaterials acting as an interface between electronics and biology. This is ascribable to the highly tunability of chemical/physical properties which confer them different conductive properties for various applicative uses (*i.e.*, molecular targeting, biosensors, biocompatible scaffolds). This review article is divided into three parts: the first one is an overview on EAPs to introduce basic conductivity mechanisms and their classification. The second one is focused on the description of most common processes used to manipulate EAPs in the form of two-dimensional (2D) and three-dimensional (3D) materials. The last part addresses their use in current applications in different biomedical research areas including tissue engineering, biosensors and molecular delivery.

## 1. Introduction

Electroactive polymers (EAPs) represent an emergent class of organic materials with intrinsic conductive properties which can be accurately controlled by modifying chemical and physical properties as a function of the specific applicative uses (*i.e.*, molecular targeting, biosensors, bio-instructive scaffolds). Their conductive behavior, similar to those of metals and semiconductors, [1] is strictly related to their highly conjugated electronic structure which is deputed to support charge mobility along the backbone of the polymer chain. 

The main advantages of these polymers rely on the ease of synthesis and flexibility in their chemical structure which allows modulating—as required—specific functionalities of materials [2]. The last dynamic and versatile characteristic of EAPs makes them suitable candidates for the development of intelligent materials with highly controllable behavior in response to external stimuli [3]. However, the high variability of EAP properties requires the fine control of them during their synthesis as a function of external stimuli (*i.e.*, magnetic, mechanic, chemical, *etc.*).

In consideration of the growing interest of the scientific community for the development of synthesis and doping strategies for EAPs, we propose an overview of current trends on the use of EAPs in biomedical applications, mainly correlating the chemical features of different EAP classes to their specific functionalities. This approach aims at suggesting the most appropriate conductive polymers/processes to be used for the fabrication of on-demand systems (*i.e.*, scaffolds, biosensors, drug delivery systems) as a function of the specific applicative demands.

### 1.1. Mechanism of EAPs Polymers: Conductivity Source and Doping

The source of conductivity in EAPs relies on the characteristic electronic structure of the extended π-bound system, consisting of a series of alternating single (σ) and double (π) bonds along the polymer chain [4]. The planar arrangement and the alignment of the *p_z_* orbitals allow overlapping between the two double bonds. The unpaired electrons (*i.e.*, π-electrons or one of the unbonded *p*-electrons of the heteroatom) are delocalized over the full length of the polymer backbone [5]. Since the electrons in the π-bonds are less strongly bonded than the electrons in the σ-bonds, they can be more easily removed from the bonds, thus favoring chemical attacks. This also suggests that conjugated compounds have the potential to display either semiconducting or metallic behavior [6]. 

**Figure 1:**
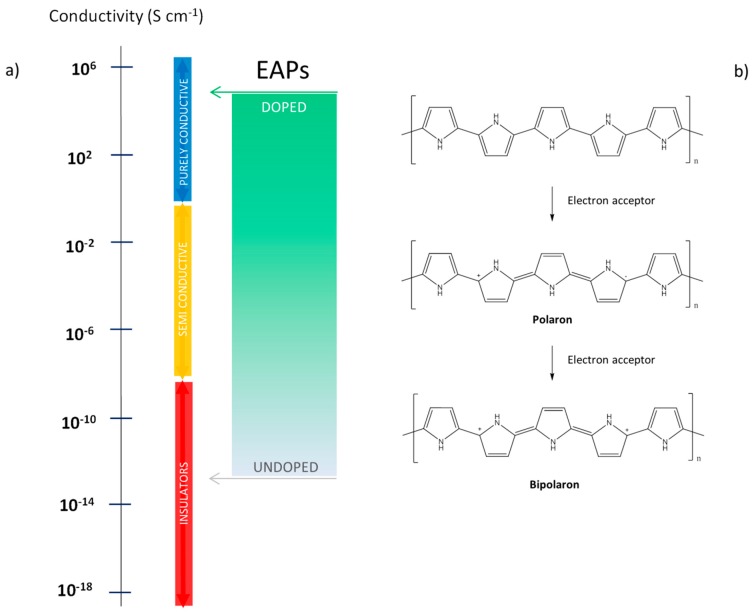
EAP properties: (**a**) Conductivity range of conducting polymers and polymer-based composites (inspired from Kaur *et al.*, 2015 [8]); (**b**) Polaron and bipolaron formation upon oxidation (p-doping) of polypyrrole.

The electrical conduction and, thus, the ability of a material to pass current defines the conductivity of a material. Based on electrical conduction measured at 25 °C, materials were generally classified in: insulators, semiconductors and conductors [7] (Figure 1a).

The extended conjugated system provides a semiconducting characteristic to conjugated polymers, which can be enhanced by the so-called “*doping*” process, in analogy with what occurs for semiconductors. It is well known that conducting polymers are not usually conductive in the basic form and doping of the π-conjugated backbone results in a highly conducting state of the polymer. By doping conjugated polymers it is possible to increase the electrical conductivity of the material, and in some cases by several orders of magnitude (*i.e.*, from 10^−10^ to 10^3^ S∙cm^−1^) [9]. An easier understanding of doping-induced changes in electronic structure can be obtained by referring to the *band theory* [10]. It is noted that the energy difference (E_g_) between the highest occupied band (valence band) and the lowest unoccupied band (conduction band) is the band gap indicative of the conductive character of material. The reduction of the E_g_ enhances the access to the conduction band, thus increasing the number of charge carriers [11]. This behavior is provided by doping conjugated polymers though a hopping mechanism mediated by dopant molecules to polymer chains. In particular, the extraction of electrons from the valence band (p-doping) generates ‘positive charged holes’ in the electronic structure, while adding electrons to the conduction band (n-doping) generates a negative charge. In both cases a possible charge mobility of electrons is created, so the conductivity is enhanced. Differently from inorganic semiconductors, conjugated polymers have a structure flexibility which allows them to locally distort to neutralize the generated charge. The introduced local charge defects in polymers are identified as polarons, bipolarons and solitons depending on the nature of the deformation [12]. In particular, when an electron is removed (or added), a radical cation (or anion) called a polaron is created, and it can be converted into a bipolaron if a second electron is removed (added). In Figure 1b the scheme of polaron and bipolaron formation upon oxidation of polypyrrole (PPy) is shown. Conversely, a soliton (neutral radical) is generated in conjugated polymers with degenerate ground states such as *trans*-polyacetylene. In order to preserve charge neutrality, a mass transport between the polymer and an electrolyte due to a variation of ionic charge is required [13]. Thus, once a charge is generated in the polymer backbone, a counter-ion acting as a dopant must be present to balance the total charge. When the polymer is oxidized (owning a positive charge) it incorporates anions, and when reduced (owning a negative charge) it incorporates cations or expels anions [14].

Among several factors influencing the conductivity of polymers, the type and concentration of dopant and synthesis methods are the most incident [7,15,16]. For p-doping, which is mostly performed on EAPs, several dopants are commonly used such as strong inorganic hydrochloric acid (HCl), aromatic and sulfonic acids, and amphiphilic dopants containing different aromatic substitutions. Comparative studies on the effect of different types of dopants on the properties of polyaniline (PANI) have been reported. In the conductive emeraldine form, PANI was generally doped by exposure to protonic acids. Studies on different protonation percentages (between 10% and 90%) demonstrated that as the doping level is increased, the conductivity increases and becomes more weakly dependent upon the temperature [17]. By choosing a suitable dopant, other important properties of EAPs such as solubility, processability, and moisture absorption can be improved. For example, in order to process the poorly soluble PANI in organic solvents, an organic aliphatic acid with a long chain length (*i.e.*, lauric acid) can be used. Alternatively, aromatic dopants reduce the moisture adsorption in PANI, probably due to the bulky size of the rigid benzene ring that hinders the bond formation of the water molecule with the back-bone nitrogen [18]. On the other hand, these larger dopants cannot increase conductivity. Hence, the choice of the more appropriate dopant to be used is a function of the material’s required demands. 

The doping process can be realized through various methods: chemical doping, electrochemical doping, photo-doping [19], charge injection doping [20,21], non-redox doping and secondary doping [22]. The process of doping occurs after and/or during the synthesis of conductive polymers, and the most widely used techniques are chemical and electrochemical methods. Synthesis conditions (temperature, solvent and water content, oxidation and pH environment, *etc.*) have a strong effect on the conductivity and properties of polymers (*i.e.*, mechanical properties and surface morphology). This does not always allow us to obtain reliable and reproducible chemical synthesis [23,24]. Hence, electrochemical synthesis is often preferred because it offers easier procedures [25], allowing the preparation of a series of doped, conducting polymers, free-standing homogeneous and self-doped films, and thus copolymers and grafts. It is worth noting that doping serves to improve not only the electric performance, but also the mechanical manageability of the material [26], which, when unmodified, is insoluble and hardly processable due to its rigid conjugated structure. Doping processes commonly promote the formation of positively charged polymer chains by monomer oxidation. So, anionic molecules are required to impart polymer charge neutrality. The conductivity of a polymer is strictly dependent on the size and amount of dopant used. The type of dopant required to be incorporated in the polymer can drive the choice toward the more appropriate synthesis method. Indeed, as an example, molecules that are not capable of redox reactions should be incorporated in the polymer by electrochemical synthesis. Conducting polymers can be reversibly doped and undoped thanks to their electronic structure, and this offers relevant benefits in drug delivery applications. Indeed, applying an electrical potential, a small dopant (*i.e.*, Cl^−^) can be induced to leave or re-enter the polymer [27], thus providing changes in volume, which expands/contracts in mass transport [28]. Alternatively, this behavior does not occur for larger dopants which tend to stably reside in the polymer backbone, forcing at least an external positive charge (cation) to enter the polymer [29]. Larger dopants affect the polymer surface and bulk properties more drastically. 

A wide variety of dopants have been used in biomedical applications, ranging between the simplest salt ions to negatively charged molecules such as polysaccharides, aromatic sulfonic acids [30], peptides, polymeric anions [31,32,33]. Independently of their biological activity, the inclusion of doping agents may influence the characteristic physical properties of the final polymers. By choosing an appropriate dopant, the properties of conducting polymers can be tailored to a specific application or, additionally, these properties can be altered and controlled through stimulation (e.g., electricity, light, pH) even after synthesis [34,35]. This aspect will be discussed in more detail in next sections.

### 1.2. EAP Classification

**Figure 2:**
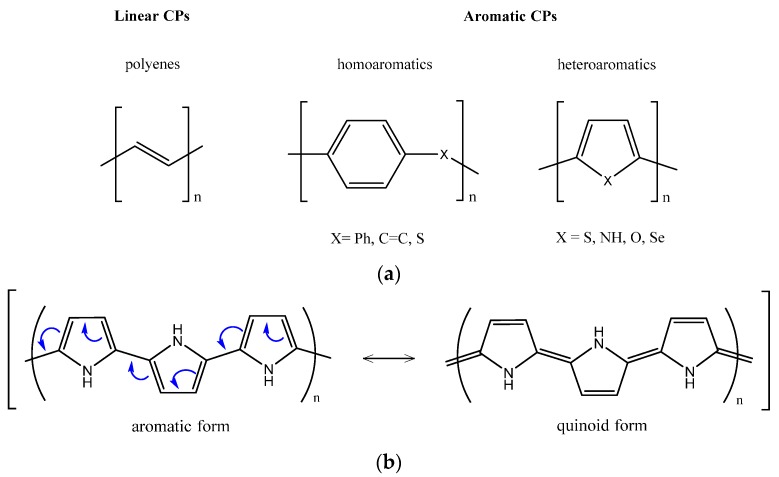
(**a**) Basic classification of conjugated polymers (CPs); (**b**) Mesomeric limiting aromatic and quinoid forms—case of PPy.

The broad range of different EAPs currently offers over 25 reported conducting polymer systems [36]. The conjugated structure, consisting of an extended π-bonded system with alternating single and double bonds, is common to all conducting polymers. They can be simply classified in linear and aromatic (homo- and heteroaromatics) conjugated polymers, whose basic backbones are shown in Figure 2a.

Homoaromatics mainly possess a benzene ring in the monomer unit, while heteroromatics consist of five membered rings, which contain heteroatoms bridging C1–C4 atoms of the *cis*-polyaromatic backbone. Furthermore, differently from linear and homoaromatic polymers, heteromatic ones consist of two mesomeric limiting forms, aromatic *vs.* quinoid, that are not energetically equivalent (Figure 2b). Heteroatoms play an important role in determining the band gaps of the conjugated polymers and are responsible for the higher stability of the aromaticity in the quinoid forms [37,38]. The results of Valence Effective Hamiltonian (VEH) calculations for polythiophene (PTh) predict that the band gap decreases as the quinoidal contribution increases [12]. This aromatic character results in competition between π-electron confinement within the rings and delocalization along the chain.

As described in the previous section, tunable electrical conductivity can be realized by several orders of magnitude via the doping process. Actually, polyacetylene (PAc), the simplest and most representative of the linear conjugated polymer class, results in the most conductive polymer, but it is unfortunately difficult to synthesize and process and it is not stable in air. Hence, the search for alternative conducting polymers was necessary. Depending on several factors such as topology or chemistry (*i.e.*, side groups or the heteroatomic substitution), aromatic polymers can achieve good maximum conductivity values, but they are generally lower than that of PAc [7,12]. In Table 1 some of the most commonly used EAPs in biomedical applications are reported correlated to the maximum conductivity values which can be measured in doped forms. The reason for this behavior relies on the characteristic electronic structure. In the homoaromatic polymers, the interaction of the bridging groups with the π-system is very weak, so the electronic effect of the bridging groups on the *band gaps* is quite small. Due to this weak interaction, the bond-length alternations of these polymers slightly change in comparison with those found in PAc. However, it is worth underlining that non-degenerate ground states of aromatic systems have important consequences regarding electrochemical synthesis (*i.e.*, favored one-step procedures of synthesis/doping) and the nature of the charged species involved in the mechanisms of charge transport [11]. These important aspects, related to good processability, combined with major stability (*i.e.*, in air) and represent a proper compromise with conductivity. Indeed, aromatic polymers such as PPy, PANI, PTh and its derivatives such as poly(3,4-ethylenedioxythiophene) (PEDOT) are widely explored in biomedical applications [4].

## 2. EAPs Processing

The main approaches to designing EAPs in materials science involve the optimization of electrochemical processes—with one or multiple steps—to directly manipulate polymers in the form of thin layers (*i.e.*, film or coating). However, electrochemical synthesis is often limited by the use of monomers which must be oxidized to form reactive radical intermediates or ions to trigger the polymerization. Despite several novel conducting polymers with selected monomer inclusions are only processable by chemical polymerization, commonly used EAPs (*i.e.*, PPy, PANI, PTh) can be polymerized both chemically and electrochemically.

**Figure 3:**
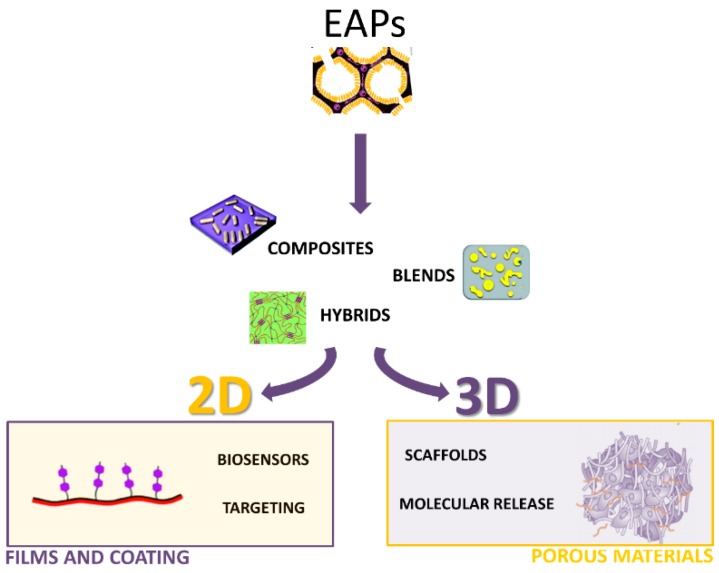
Scheme of EAP processing modalities to fabricate 2D or 3D devices for different applications in the biomedical field.

The growing demands in the biomedical field to develop highly complex systems able to match the innate structural hierarchy of biological tissues are inspiring different technological solutions for processing virgin conductive polymers, as “undoped” or “doped” forms, by controlling their morphology at different size scales. They mainly involve the chemical (*i.e.*, polymerization) or physical (*i.e.*, thermoplastic processes) manipulation of EAPs to combine one of the different polymers to fabricate blends, composites or hybrids in the form of coatings or porous materials (Figure 3). Here, a brief overview of current technologies implemented to design EAPs using different chemical-based technologies as a function of the peculiar morphology of the final device is reported.

### 2.1. Two-Dimensional (2D) Processes for Coatings

In order to fabricate polymer films with controlled thickness, several techniques have been proposed over the years. The solution cast and spin cast methods, depending upon the solubility of the conducting polymers (previously synthesized by chemical polymerization), can be used for the fabrication of ordered films. The first method consists of depositing drops of monomer and oxidant solution on the substrate surface and obtaining the film with the evaporation of the solvent. This process is easy and ensures that polymerization occurs exclusively at the surface, but as expected, the uniformity of the film cannot be controlled. Alternatively, by spin-coating deposition the solution of the polymer (dissolved in an appropriate solvent) is spread on the rotating substrate, and after evaporation of the solvent, a thin layer of polymer is obtained. The film thickness depends on the solution viscosity, rotation rate and duration. The Langmuir–Blodgett (LB) film deposition technique has been applied to organic thin film fabrication arranged in ordered states. Since each run corresponds to one monolayer, a fine control of molecular layer thickness is firmly required for the fabrication of LB films where the possibility of adding specific functionalities at the molecular level during film processing may represent an interesting improvement of this technique [41]. 

EAPs may contribute to functionally improve the interface between synthetic and biological tissues, providing a more conductive environment for cell integration [42] through an efficient transfer of electrical *stimuli* in electrosensitive tissues (e.g., nerve, myocardium) [43]. In addition to being electroactive, they offer the advantage of easily tuning the thickness of electrodeposited films. In this context, PPy possesses many excellent qualities and stimulus-responsive properties, which make it a very promising ‘‘smart’’ biomaterial. Optically transparent PPy thin films were synthesized to study the behavior for mammalian cell culture by Shastri [44]. Results have shown that PPy can alter protein adsorption as a function of the dopant anion and polymer oxidation state. Most importantly, it has good *in vitro* and *in vivo* biocompatibility, good chemical stability in, for example, air and water, and reasonably high conductivity under physiological conditions [45]. In the case of electrode interfaces with neural tissues, conductive polymers may enhance surface interactions [42]. Indeed, their coatings can serve to improve the electrode-tissue communication through providing a high surface area material more conductive to cell and tissue integration. However, when film is obtained by chemical techniques, it often requires a post-fabrication doping to increase conductivity. In this case, the dopant is electrodeposited by addition to the electrolyte solution from which the polymer is synthesized. Otherwise, electropolymerization without any further processes is a simple and reproducible technique to improve electrode coatings for neuroprosthetic applications [44]. In particular, PPy films were used to electrically stimulate PC12 cells and the promotion of neurite outgrowth from the cells was observed, demonstrating their potential use for the fabrication of electroactive scaffolds in nerve tissue engineering [46,47]. An interesting example consists of PPy microchannels, which were electrochemically synthesized to fabricate electroconductive, topographical substrates for neural interfacing, and results showed that the device facilitated axon establishment of rat embryonic hippocampal neurons [48]. PPy coatings were used for neural probes and to promote regeneration mechanisms (ECM growth and proliferation) in damaged rat peripheral nerves [15]. *In vitro* experiments demonstrated that these films supported attachment and growth of all endothelial cells. However, flat substrates obtained by these techniques do not represent the best model to study cell responses to electric fields, but a three-dimensional (3D) architecture is required to promote the re-organization of a functional nerve tissue. PPy can be easily and flexibly synthesized in large quantities at room temperature in a wide range of solvents, including water [49], but, unfortunately, PPy is very difficult to further process once synthesized. PANI offered a valid option to induce electroactivity in biomaterial scaffolds and substrates, and its popularity has attracted considerable attention in biological systems due to its support of cell growth. For example, PANI implants were tested in different oxidation states (emeraldine, nigranidine and leucoemeraldine) *in vivo* in order to assess the tissue response. Results demonstrated the absence of significant inflammation at the implant sites [50], further proving that PANI did not cause inflammatory responses in the subcutaneous tissues over two years [51]. Recently, Bidez *et al.* also found that both PANi in different forms, *i.e.*, non-conductive emeraldine base and conductive salt forms, allows promoting cell attachment and proliferation [52]. PANI is generally synthesized by either chemical or electrochemical methods [53]. Electrochemical methods including potentiostatic (constant voltage), galvanostatic (constant current) polarization and cyclic voltammetry allow obtaining a highly pure and uniform PANI layer suitable for metal electrode coating [54]. Chemical methods to synthesize PANI are commonly based on the polymerization of monomers by oxidation in aqueous solution, while others consist of emulsion, dispersion, solution, interfacial, metathesis, and self-assembling polymerization [55]. However, once synthesized, PANI is difficult to use in its pure form in biological systems for practical application because it is generally nonflexible, non-processable and non-biodegradable. The use of electrodes for neural prostheses has been growing in recent years, and accordingly, continued attempts to improve their electrical properties and biocompatibility have been based on the development of conductive polymer coatings. Processes at the interface between the electrode and electrolyte, which are involved in the physiological environment, have driven considerable experimental and theoretical attention to increase the surface area of the electrodes. The interest in interfacial contact between the electrode and the tissue is due to a need for promoting cell adhesion and also facilitating charge transport [56]. Neural probe coatings are generally fabricated by the simultaneous precipitation of conductive polymers and biocompatible anions at individual electrode sites. However, the major limitations of this technique consist of the lack of high control of films’ structural properties at the micrometer or nanometer scales, and of the weak attachment of the coating on metal. In this context, various attempts were made to improve an example consisting of the electrodeposition of heteroaromatic conducting polymers in a novel solvent system based on trifluoroborate-ethyl ether (BFEE). Widge *et al.* have proposed an alternative to electrodeposition, developing self-assembled monolayers (SAMs) of organic molecules [57]. This new type of coating is based on mixed thiolated poly(alkylthiophene)s and functionalized alkanethiols. This process allows tailoring of the film properties at each electrode.

Keeping in neural electrode applications, another very interesting conjugated polymer is poly(3,4-ethylenedioxythiophene) (PEDOT), a polythiophene derivative. It showed higher electrical, thermal and chemical stability than PPy [58]. Furthermore, a neural electrode was interfaced with the surrounding brain tissue through the *in situ* polymerization of PEDOT [59]. It has also been polymerized within a cellular muscle tissue, where it formed a network of elongated tubular structures throughout the tissue [58], in essence converting it into an extensive conductive three-dimensional substrate. However, it is still desired for these and other applications to provide additional cues such as biomolecules in conjunction with electroactivity provided by the conducting polymer. These interactions may be further enhanced by the inclusion of specific functionalities or moieties on the backbone. The addition of bioactive factors to conducting polymers allows fabricating hybrid materials with improved conductive properties in terms of cell-to-cell and cell-to-materials interactions. 

### 2.2. Three-Dimensional (3D) Processing

#### 2.2.1. Blends

In the last 10 years, blending methods have represented the most efficient strategy to reach the standpoint of large-scale production and processability of EAPs. Conducting polymers blended with insulating ones may encourage a more efficient distribution of the electro-sensitive molecular groups, by reducing chemical toxicity and guaranteeing a good transfer of electrical signals, with relevant effects on cell behavior *in vitro* and *in vivo* [60]. To date, several blends have been prepared by combining degradable polymers with conducting ones in different environmental conditions. Overall, fabrication processes based on chemical blending can be distinguished in two different groups related to melting or solvent dissolution of polymers. Polymer blends via melt processing are generally used to design conductive systems for industrial scale. They are commonly synthesized through the mechanical dispersion of conducting polymers in thermoplastic melt to obtain fully moldable and extrudable systems [61]. For instance, Zilberman *et al.* have developed a conductive blend by mixing polyaniline-*p*-toluene sulfonic acid (PANI-*p*TSA) with thermoplastic polymers. The peculiar interaction between doped polyaniline and polymer matrix allows imparting the final blend morphology and, consequently, their peculiar conductivity. This mechanism is strictly related to PANI solubility and polymer matrix crystallinity which together concur to improve the physical dispersion of conductive phases into the matrix, assuring the formation of percolative conducting paths at lower PANI contents [62]. Alternatively, all the technological approaches based on EAP dissolution in different solvents are generally based on specific chemical reactions which mainly require the use of group substitution along polymers chains to promote the oligomers’ solubility in organic solvents [63]. In several cases, a doping reaction is also required via protonation of undoped EAPs by functionalized protonic acids, such as camphor sulfonic acid (CSA), dodecylbenzene sulfonic acid (DBSA), or phosphoric acid diesters which are compatible with nonpolar or weakly polar organic solvents [64].

Recently, several studies have been delivered about the design of bicomponent blends with intrinsic conductive properties for biomedical applications. In particular, EAPs such as PPy and PANI have been generally blended with biodegradable synthetic polymers, *i.e.*, poly(d,l-lactide) (PDLLA) [65], poly(caprolactone) (PCL) [66], or polyvinyl alcohol [67] or natural proteins including collagen or gelatin [68], to realize micro/nanostructured platforms for tissue engineering. It is noteworthy that the physical mixing generally improves EAPs’ properties in terms of surface charge, wettability and conformational/dimensional changes, and it is able to influence chemical or electrochemical oxidation or reduction mechanisms at the scaffold surfaces with relevant effects on cell culture [69]. Moreover, other relevant properties including biodegradability, mechanical properties and non-solubility can be influenced, with respect to the use of conducting polymers [70]. Qazi *et al.* recently proposed the preparation of a blend of PANI with poly(glycerol-sebacate) (PGS) to realize electrically conductive composite cardiac patches via the solvent casting method [60]. They demonstrated that electrical behavior switches from an insulant (*i.e.*, ~10^−11^ S∙cm^−1^) to conductive (*i.e.*, 0.018 × 10^−2^ S∙cm^−1^) state as the PANI content increases, also reporting an improvement of elastic modulus, tensile strength and elasticity. These values fall in the physiological range of native myocardial tissue, *i.e.*, E-modulus of PGS, 0.025–1.2 MPa; E-modulus of human myocardium, 0.02–0.5 MPa, thus mimicking the mechanical stiffness of the final patch. Moreover, the release of acid dopants from PGS matrices due to the presence of PANI may alter local pH, promoting a buffering effect which reduces the *in vitro* toxicity of the scaffolds favoring their normal growth and function. 

However, one of major problems still remains the preservation of conductive properties during their processing. Blending strategies may partially compromise the final conductive properties of the device [71]. This aspect has been remarked on in the fabrication of bicomponent fibers by electrospinning where electric forces strongly interact with polar groups of conductive phases [72]. In this case, exceeding applied voltage may alter fiber morphology in terms of fiber diameters, the conductive phase spatial distribution along fibers with a consequent loss of percolative organization of EAPs and, ultimately, the drastic decay of conductive properties [73,74].

#### 2.2.2. Composites

When an electrically conductive phase is dispersed in a polymer matrix, a conductive composite is formed. In this case, mechanical, thermal and electrical properties of the composites increase the local interactions between EAPs and the polymer host matrix as a function of the spatial distribution of EAPs and of their chemical/physical interface with the neighboring polymer matrix. Indeed, the influence of the polymer matrix is crucial to define the conductive behavior of EAP-based composites. In particular, internal stresses can be generated at the interface between dispersing phases and the polymer matrix as a function of local shrinkage or thermal expansion, thus ultimately influencing the conductivity properties of the composite [75]. Different biomaterials, including soft elastomers, biopolymers, rubbers, and linear or branched thermoplastics, have been variously investigated to evaluate how local contact forces exerted by the matrix on EAP phases—and *vice versa*—might influence the final conductivity of the composite. In tissue engineering, this aspect becomes really interesting for investigating the basic mechanisms of cell interaction mediated by conductive phases in *in vitro* cultures. In all these cases, conductive polymers have been used as filler or matrices to design electroactive composites for different biomedical uses. The most common strategy involves the combination of conductive matrices with inorganic fillers. In this case, the reduced biocompatibility of polymer matrices mainly leaves them for external use—*i.e.*, in all the biomedical applications not requiring the direct contact of materials with cells (e.g., biosensors, actuators, prostheses). In this case, EAPs have been generally processed by chemical (*i.e.*, oxidation) and electrochemical methods in combination with consolidated polymer manufacturing techniques (*i.e.*, emulsion, phase separation) to control the final morphology of the device. Conversely, a relevant part of the current studies mainly focuses on the development of biocomposites including conducting phases in the form of a dispersing phase along with non-conducting matrices to improve the functional performance (*i.e.*, mechanical properties) and biocompatibility of scaffolds [76]. The use of EAPs such as PPy and PANI in the form of conductive fillers allows us to overcome relevant shortcomings in terms of processability of non-polymeric conductive fillers (*i.e.*, graphene, carbon nanotubes), thus imparting more homogeneous conductive properties to insulating polymer matrices [77]. For instance, Sarvari *et al.* proposed the combined use of hyperbranched aliphatic polyesters (HAPs), PANI, and PCL to develop novel elastic, porous and conductive scaffolds for tissue engineering applications [78]. In this case, HAPs and PANI were separately synthesized by a melt polycondensation reaction while macromonomers/aniline monomer copolymerization were synthesized via both chemical and electrochemical oxidation methods. Then, they were mixed in a solution with PCL to produce uniform conductive nanofibers. Alternatively, Borriello *et al.* proposed the preparation of different composite substrates made of ultrafine polyaniline short fibers (sPANI) in a PCL matrix to be used as platforms for cardiac tissue engineering. In this case, conductivity is also generated by the peculiar morphology of dispersed fillers, characterized by needle-like crystals of PANI fabricated by *in situ* synthesis via a rapidly-mixing reaction with the initiator solution, *i.e.*, ammonium peroxydisulfate in CSA buffered water [68]. It is well known that PANI is initially non-conductive, but may be converted to the conductive state by increasing the protonation degree by acidic dopant treatments able to convert imine groups of the emeraldine base to iminium [79]. In this work, it is proved that synthesized polyaniline (sPANi) short fibers confer to the composite a strong force of charge transport, in comparison with that of emeraldine base polyaniline (EB-PANI) simple blending as a function of the selected conducting filler amount, shape and its spatial distribution. This is confirmed by conductibility tests indicating a more efficient transfer of electric signal associated with the peculiar spatial organization of sPANI short fibers forming a percolative network.

#### 2.2.3. Porous Materials

In the physiological studies of electroactive tissues (*i.e.*, nerves, muscles), the electromechanical coupling of cells is crucial for supporting the synchronous response to electrical signals [80]. However, the use of non-conductive or poorly conductive materials as cell-carrying scaffolds does not allow the conduction of natural electrical impulses, thus preventing effective cell to cell communication [81], and also drastically limiting the ability to face natural contractions of native tissue. In this context, Baeirahei *et al.* successfully fabricated electroactive scaffolds with interconnected pores and controlled porosity by a process of particulate leaching and compression molding. In this case, technological improvement mainly referred to the effect of co-porogens—NaCl crystals and PEG powder together—to enhance the cohesion of conductive phases along the final porous network. The resulting electroactive structure made of polyurethane and PCL matrix incorporating aniline pentamers with polar moieties shows mechanical and chemical properties suitable to support cardiomyocyte proliferation without external electrical stimulation for the repair of damaged heart tissue. In particular, this is confirmed by the overexpression of specific genes typically involved in muscle contraction and relaxation (troponin-T) and cytoskeleton alignment (actinin-4) [82].

More recently, Guarino *et al.* proposed the fabrication of macroporous hydrogels with controlled pore morphology and conductive properties to support cell signaling during nerve regeneration. The 3D hybrid system is prepared by *in situ* precipitation of PANI in polyethyleneglycol diacrylate (PEGDA) solution, then crosslinked via UV irradiation [1]. The porous architecture, characterized by macropores ranging from 136 to 158 μm in size, has been achieved by mixing/removal of water-soluble inorganic crystals. This approach based on PANI chemical synthesis and hydrogel manufacturing by consolidated scaffold preparation techniques allows designing innovative EAP materials with suitable conductive behavior and biocompatible features for *in vitro* studies. Indeed, the presence of PANI evidently imparts the desired electrical conductivity to the hybrid material—1.1(±0.5) × 10^−3^ S∙cm^−1^ for just 3 wt % PANI content—without relevant cytotoxic response. PANI phases in the presence of large macropores improve water retention, thus promoting a more efficient charge transport mechanism. Meanwhile, their innate hydrophilic properties—homogeneously exerted by the 3D matrix—remarkably contribute to greatly influence proton conductivity, assured by the coordination of high amounts of water molecules in the polymer matrix which support the proton exchange reaction. In this context, the full interconnection of the macropore network assures uniform cell colonization as well as the homogeneous growth of newly forming tissue. This is directly ascribable to the full percolation of fluids through the hydrogel matrix supporting a correct nutrients/waste exchange as well as a more appropriate supply of molecular signals required to trigger early and late cellular events. Moreover, the local biological microenvironment also contributes to the ion transport mechanisms, supplying the transfer of the electrical signals mediated by porous scaffolds to cells in *in vitro* culture. All these relevant properties play an important role in addressing cell activities as confirmed by the biological response of PC12 and hMSC cells. Hence, they conclude that the mutual contribution of hydrogel scaffold functionalities, *i.e.*, porosity and conductive properties, represents a fundamental pre-requisite to drive nerve cells in regenerative processes. It is noteworthy that this effect is evidently amplified by the PANI content increase, which determines a relevant reduction of local distance among fibers, with consequent enhancement of tunneling or hopping processes.

Starting from the previous study, novel porous conductive hydrogels based on gelatin and/or chitosan have been recently designed for tissue engineering. For example, the combined use of organic (*i.e.*, PPy) and inorganic (*i.e.*, graphene) conductive phases has been investigated in terms of functional properties of the scaffolds. In particular, by introducing polypyrrole/graphene (PYG) into the polymeric matrix, the porosity and swelling capacity decrease while electrical conductivity and Young’s modulus conversely increase. Meanwhile, *in vitro* biodegradation tests present a radical reduction of the biodegradation rate in the presence of PYG conductive phases with respect to the gelatin/chitosan matrix alone, with 80% weight loss after six weeks due to the lysozyme effect. This peculiar behavior seems to not affect Schwann cell attachment and proliferation for a PYG amount not exceeding 10%, under electrical stimulation, thus it is suitable for nerve tissue engineering applications [83]. 

Similarly, poly(3,4-ethylenedioxythiophene)poly(styrenesulfonate) (PEDOT:PSS) has been templated into 3D macroporous structures to form electrically conductive, low-impedance, and biocompatible scaffolds for fibroblast cultures [84]. Scaffolds have been fabricated by ice-templating, a versatile technique based on polymer solution freezing with the subsequent confinement of solute particles in the regions where solvent crystal dendrite formation and ice crystal sublimation occur [85]. This technique may be optimized by combining different removable templates to generate porosity at multiple length scales by a single processing step [86] to obtain different structures including lamellar, “fish-bone”, aligned tubes, and cellular foams [87,88], with peculiar conductive properties [89]. Wan *et al.* recently demonstrated that fibroblasts seeded onto the scaffold remain viable for seven days, showing regular cell functions such as adhesion and deposition of ECM components [79]. Moreover, electrochemical stimulation of the scaffolds leads to controlling the assembly of adsorbed ECM proteins (*i.e.*, fibronectin) with the opportunity to influence adhesion and pro-angiogenic cell secretion. Other studies demonstrated that the low impedance of macroporous PEDOT:PSS scaffolds makes them suitable to be integrated into transistors for sensing applications [90], in order to detect real-time signals from cells cultured within the scaffolds. Hence, these preliminary studies on macroporous EAP scaffolds may be relevant to designing *ex novo* electrically active, physiologically relevant 3D tissue culture platforms to guide and control cell activities (*i.e.*, regenerative medicine) but also to monitor cell functions in changing microenvironments (*i.e.*, biosensing).

## 3. Applications in Biomedical Field

### 3.1. EAPs to Guide Cell Functions: Tissue Engineering

The growing interest for electric/magnetic stimulation in the medical field arises from a growing knowledge of the intrinsic functionalities of living tissues [91]. Natural tissues generate electromotive forces, maintain a required potential gap, and switch current on and off by controlling current flow and store charge [92]. It is well known that electrical voltage exists across the plasma membrane, while the inner part of cells results is more negative than the outer part. This potential gap, namely resting potential, is maintained at a steady level when excitable cells are inactive [93]. Hence, the application of electrical stimuli may strongly influence cell activity in terms of intracellular signal transduction pathways, due to the alteration of membrane potential associated with transmembrane ion transfer mechanisms [94]. In this context, the use of electrical signals to control the local microenvironment for cells may be crucial to trigger basic activities towards specific phenotypes in order to reach a long-term functionality of tissues during the *in vitro* regeneration processes [95]. In current tissue engineering practices, cells are generally forced to survive in a dynamic environment reproduced by custom-made scaffolds which mimic the composition and topography of a natural niche, by the support of physical factors, such as mechanical and electrical signals [96]. 

**Figure 4:**
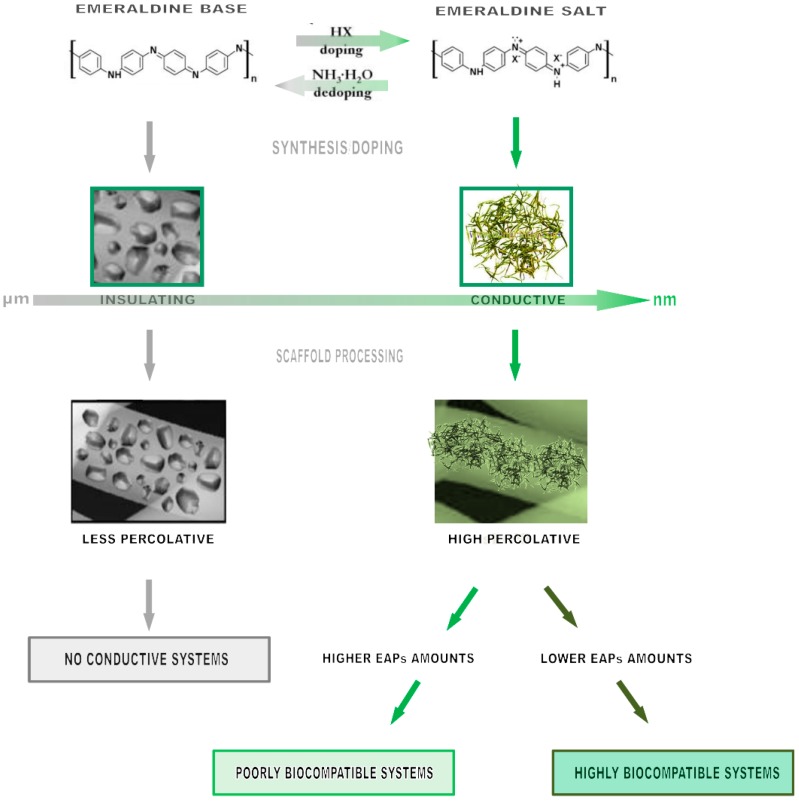
Schematic description of the biocompatibility of conductive platforms including EAPs: How conductive phase morphological features (*i.e.*, shape, amount) can influence the transfer mechanisms of electrical signals.

Suitable materials from polymers, ceramics and metal classes—defined as biomaterials—can be properly manipulated to mimic the structural organization of native extracellular matrix, supporting cell growth without any adverse *in vivo* reactions. In this context, the introduction of specific stimuli plays an important role in the development of *in vitro* tissues through the physical support of the scaffold microarchitecture [97]. In the recent past, several studies have been focused on the incorporation of conductive particles including carbon nanofibers [98] and gold nanowires [81] into polymer matrices to modulate cellular behavior. However, the presence of conductive elements does not assure the transmission of electrical signals without the use of external sources among cells, and this may represent a relevant problem after implantation, due to unknown long-term effects *in vivo*. Alternatively, the use of conducting polymers, in turn, dissolved in organic solvents and/or blended with other polymers, currently offers a suitable alternative to designing biomaterials for biomedical use. In particular, EAPs improve scaffold functionalities by properly supporting the electrical stimulation among cells which is mandatory for promoting regeneration mechanisms in the case of specific cells (*i.e.*, neurons, myotubes, cardiomyocites) which respond to pulsatile and abrupt electrical stimuli [99]. To date, it has been demonstrated that EAPs are able to positively influence cellular activities, including cell adhesion and migration, DNA synthesis and protein secretion [51,100]. In particular, recent studies have confirmed the efficacy of conductive polymers in stimulating a multitude of late cell functions, including proliferation and differentiation, and also maintaining *in vivo* activity in a physiological environment for significant levels of applied electrical signals [101]. This mainly depends upon the unique capability of EAPs to intrinsically transfer electrical signals to cells without the use of external devices for electrical stimulation [66]. Indeed, as a function of the conducting filler amount and related spatial distribution in the polymer matrix, the physical properties may be drastically changed. At very low filler fractions, the mean distance between the conducting phases is quite large and the electrical conductivity is mainly determined by the host matrix. When the conductive phase amount exceeds a critical value, they form punctual bridges which lead to the formation of conductive paths able to “percolatively” transfer the electrical signal (Figure 4).

A crucial aspect for the use of EAPs in tissue engineering and regenerative medicine mainly concerns how conductive properties can influence biocompatibility, hydrophobicity, and surface topography by controlling peculiar mechanisms of chemical synthesis (*i.e.*, reversible oxidation, redox stability). Recently, Hsiao and colleagues developed a conductive composite scaffold of PANI and poly(lactic *co*-glycolic acid) (PLGA) with electrospinning [102] as a conductive platform to electrically stimulate cardiomyocytes at the molecular level (*i.e.*, protein expression related to the synchronous cell beating). Similarly, other studies have reported promising results in terms of enhanced intercellular communication, improved tissue alignment, expression of cardiac-specific proteins and enhanced cardiomyocyte function after incorporating conductive elements (PANI, PPy, gold nanowires, carbon nanofibers) in scaffolds, followed by the electrical stimulation of the seeded cells [100].

Recent studies have also investigated cell compatibility on composites including PPy, the most used EAP in the biomedical context, once specific current or voltage is applied to different substrates, with a wide range of cell types, *i.e.*, myoblasts and nerve cells, *in vitro* and *in vivo* [36,59]. For instance, conducting composite conduits based on PPY and poly(d,l-lactic acid) (PLA) have been developed to properly support the differentiation of rat PC12 cells for the *in vivo* regeneration of peripheral nerves. *In vitro* and *in vivo* studies have confirmed the ability to regenerate a functional nerve similar to the autologous graft during the clinical treatment of rat sciatic nerve defect [103]. Besides, the most significant limitation of using EAPs in tissue regeneration is their inherent inability to degrade naturally. Current attempts mainly involve the coupling of EAPs with biodegradable polymers. Their peculiar composition allows combining polyester biodegradability and EAP conductivity for successful application as temporary scaffolds with electroactive functionalities. In this context, proteins and polysaccharides have been recently mixed to support and regulate cell adhesion, proliferation and differentiation *in vivo*. For instance, the use of slightly positively charged proteins such as gelatin—able to electrostatically attract the slightly negatively charged cell membrane—may support EAPs’ function to catalyze the basic mechanisms of cell attachment, thus promoting the formation of more efficient binding sites [104].

Several strategies have been investigated to “bio-molecularly” modify conducting polymers to improve their biocompatibility by covalent grafting or direct incorporation of dopant molecules. For instance, cell-adhesive peptides have been covalently grafted onto PPy to significantly improve adhesion and spreading of neonatal rat osteoblast cells and human umbilical vein endothelial cells (HUVECs) compared with unmodified PPy surfaces. Surface functionalization by stably binding short peptides has been alternatively proposed to immobilize RGD on PPy surfaces [105] to promote PC12 cell adhesion in serum-free medium. 

Some macromolecules composing native ECM, including hyaluronic acid (HA) [106], collagen [106], laminin fragments [107], and dermatan sulfate [108], have been incorporated as dopants or directly entrapped into conducting polymers to improve the biocompatibility of the final device. For instance, HA has been endowed in PPy matrices to promote the vascularization process in implanted rats [109], while laminin fragments have been combined with PPy to enhance *in vitro* nerve cell adhesion and spreading [107]. Growth factors can be also incorporated to improve the bioactivity of conducting polymers. For example, Lee *et al.* have demonstrated that nerve growth factor (NGF) linked by active ester groups may preserve their bioactivity when incorporated into PPy or PEDOT [110]. Alternatively, NGF has been also covalently immobilized via the arylazido functional group, showing negative outcomes in terms of endocytosis inhibition ascribable to a relevant decay of EAP conductivity [111]. In this scenario, the challenge to synthesize EAP with tailored requisites in terms of biocompatibility and biodegradability to minimize the inflammatory reaction in the host tissue is still opened. Recent studies are exploring the synthesis of new polymers, based on polymers with a biodegradable backbone structure, incorporating aniline units to impart the conductive properties, including PCL or polylactide grafted byaniline oligomers [112]. Alternatively, biodegradable electroactive hydrogels have been synthesized via aniline pentamer (AP) grafting gelatin (GA) [113]. In this case, hydrophobic pentamers alter the porous structure of the protein-based hydrogel without affecting cytotoxicity, which is strictly related to biocompatible properties of the gelatin structure. More recently, conductive hydrogels have been synthesized by the grafting of polyaniline and gelatin macromolecules at physiological conditions [114]. The gelation time, swelling ratio and degradation time have been easily tuned by an accurate selection of polymer ratios and genipin content, thus promoting great adhesion and proliferation of osteoblasts and C2C12 myoblast cells, for muscle skeletal tissue repair.

### 3.2. EAPs to Target Drugs and Biological Molecules: Drug Delivery

The progressive discovery of materials with new chemical functionalities (*i.e.*, magnetic [115], optical [116], piezo [117], magnetopiezo [118]) is running towards the development of novel drug delivery systems able to face new application fields (*i.e.*, diagnostic, teranostic), which were previously unsuited to traditional delivery systems. In particular, the integration of EAPs in drug delivery systems represents an interesting approach due to their biocompatibility and their possibility of to be used for real-time monitoring in *in vivo* biological environments [119]. Several years ago, EAPs had been just manipulated by electrochemical processes to control the surface dropping of protein molecules encapsulated in polymer matrices [120]. In that case, the local change of conductive polymers at the redox state allowed increasing the drugs’ diffusion, thus providing an easier exit from the polymer matrix [121]. Several subsequent studies have demonstrated that EAP-based drug delivery systems are prepared by electrochemical oxidation rather than chemical oxidation because the former allows for tighter control over the quantity and the properties of the produced polymer by modification of the synthesis conditions [122]. Indeed, during reversible redox reactions, EAPs show an alteration in redox state generating simultaneous changes in polymer charge, conductivity and volume. Hence, the rate of drug release from EAPs can be modified. Meanwhile, other parameters including electrical stimulus magnitude, anodic potential, pH and temperature conditions may drastically influence the EAP properties, thus influencing drug release [123,124]. 

In the past, electrical stimulation of EAPs has been used to release a large number of therapeutic molecules including growth factors [125], drugs [122] and mucopolysaccharides [126]. Besides, the choice of drugs to combine with EAP systems generally depends upon several factors. Firstly, they should be not electroactive at the processing and/or working conditions in order to compromise molecular efficiency and cell biological activity. Moreover, they have to be soluble in the polymer solution in order to improve the encapsulation in polymer matrices, and minimize the formation of local aggregates able to affect release history.

**Figure 5:**
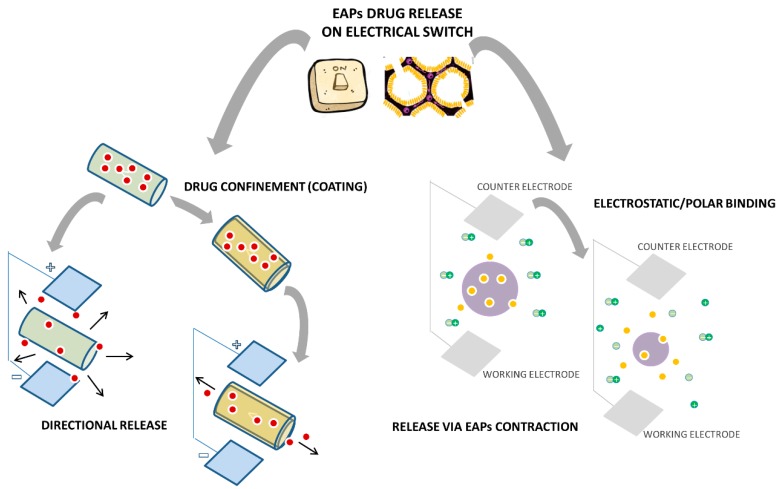
Mechanisms of “on demand” molecular release triggered by electrical stimulation of EAPs.

It is noteworthy that the peculiar molecular charge may drastically influence the process of EAP fabrication. For example, several studies have investigated the use of PSS in combination with conductive monomers and small molecules (*i.e.*, nerve growth factors (NGF) [127]. In these cases, NGF molecules acting as a neurotrophic co-dopant with net positive charge at neutral pH are electrochemically entrapped into the composite in the form of a polyelectrolyte complex, showing slower release kinetics directly related to local charge interactions, able to influence *in vivo* cell response [128]. A different mechanism may occur in the case of conductive phases with heterogeneous dispersion at the nanometric-size scale. Abidian *et al.* have investigated the use of PEDOT nanotubes polymerized onto electrospun PLGA for the release of dexamethasone. In this case, the drug was incorporated within the PLGA fibers while the polymerization of conductive polymer occurred among the dexamethasone-loaded PLGA fibrillar structure. As fibers degraded, dexamethasone molecules entrapped by PEDOT nanotubes provided a controlled release of the drug upon electrical stimuli [129]. This study is a pioneer of more recent drug delivery systems based on the “on-demand release” mechanism mediated by external electrical stimulation for innovative regenerative and degenerative medicine (*i.e.*, cancer) approaches (Figure 5). For instance, electroconductive hydrogels have been extensively studied due to the unique possibility to confine them in a sole material; the best properties of hydrogels and conducting polymers are: high water swelling, high molecular permeability, *in vitro* and *in vivo* biocompatibility, high electrical conductivity, “on-off” electrical and optical switching, electrochemical-triggered redox properties and volume changes [34]. Independent of the presence of conducting polymers, the hydrogel swelling degree is strongly affected by ionic strength. As this material is responsive to changes in the electrical potential, pH and ionic strength, molecular release presented different delivery profiles in accordance to submitted stimuli and their variations [130]. Several studies have described simple and elegant routes to obtain degradable and electrically conductive hydrogels (DECHs) by *in situ* synthesis, overcoming major limitations in the poor processability of commonly used conducting polymers [131].

More recently, several efforts have been focused on engineer stimuli-responsive EAP devices with customized release of therapeutic molecules according to specific cellular or extracellular stimuli triggered via chemical, biochemical, or physical means. During the release mechanism, several changes in the nanocarrier structure or chemistry may occur, which drastically influence the release of the therapeutic payload as a function of the peculiar features of carriers and their interaction with biological environment stimuli. Accordingly, a recent study describes the on-demand, electronically controlled loading and Ibuprofen sodium salt release from a highly porous PPy layer onto carbon cloth electrodes [132]. In this case, all drugs are properly released upon application of either an oxidizing or reducing potential as a function of the peculiar polymer and drug polar groups. However, the opportunity to manipulate the release properties of EAP-based matrices from outside the body may involve the interaction with several “exogenous *stimuli*” including heat, light, electrical, magnetic or ultrasound signals [133]. This opens a real challenge to designing “on-demand release systems” able to minimize systemic toxicities and unfavorable drug-plasma interactions, in order to dose and more efficiently treat pathological, chronic or traumatic diseases 

### 3.3. EAPs to Monitor Cell Activity: Biosensors

A biosensor can be generally defined as a device consisting of a biological recognition system (often called bioreceptor), and a transducer [134]. The bioreceptor (biomolecules such as enzymes, bacteria, microorganisms, cellular receptors, antibodies, nucleic acids, *etc.*) is a biological sensing component able to specifically interact (by binding or recognizing) with an analyte, producing a biochemical effect. The transducer converts this effect into a measurable signal (*i.e.*, electrical or optical). In electrochemical biosensors, the transducer produces an electrical signal generated from electrochemical reactions occurring at the electrode-solution interface. 

In the field of biosensors, the use of bioactive surfaces is of particular interest, due to the strong interaction of biological systems with biomaterials at the interface. So, a large variety of chemical modifications/immobilization methods have been developed to encode bioactive ligands able to selectively interact with specific cell phenotypes. 

EAPs represent a valid choice as biosensor materials thanks to suitable advantages: efficient electric charge transfer from bio-reaction (due to the electronic structure of extended π-bonded molecules), electrochemical synthesis *in situ* on electrodes, the ability to entrap biomolecules, possible surface modification [135]. The earliest studies on conductive polymers in biomedical applications focused on the development of new bio-accepted materials suitable for biosensor applications [51,136]. Their electronic properties also permit transfer charge between the incorporated biomolecules and the electrode to produce a range of analytical signals, thus acting as an electronic transducer for charged species binding to their surface. EAPs can be used for modifying electrodes in polymer-based systems because of the possibility of being deposited on metal electrodes. This procedure under mild conditions allows entrapping biological molecules (*i.e.*, enzymes, antibodies, growth factors, DNA or RNA fragments) or other reagents able to trigger biochemical reactions at the electrode interface [137].

Among EAPs doped and/or covalently or not covalently modified by bionanomaterials, PPy, poly(*N*-methyl pyrrole), polyindole, PANI, polycarbazole, PTh and derivatives (*i.e.*, PEDOT) exhibit unique catalytic or affinity properties that can be easily applied in the design of biosensors [138,139]. 

Several important properties are required in the design of electrochemical biosensors based on EAPs: mechanic stability and electrical conductivity, which are provided by the substrate electrode (*i.e.*, Pt, glassy carbon, Au, SnO_2_, metallized plastics, carbon fibers, or TiO_2_), conducting properties of the polymeric matrix for immobilization, and the immobilized biological component [140]. Actually, the most important required aspect is an efficient and effective immobilization of the biological moiety on the substrate electrode. Entrapment of these bioreagents can occur through different procedures: electrochemical immobilization, physical adsorption, affinity binding, covalent linking, cross-linking [25]. The main strategies involve the molecular absorption, covalent bonding or polar bonding during the electrochemical process [141]. Obviously, the choice of which procedure is most appropriate is based on the necessity to retain the chemical and electrical properties of both EAPs and biomolecules that are immobilized [134]. Biomolecule entrapment—a versatile strategy largely used to fabricate biosensors—consists of a one-step procedure involving the application of an appropriate potential to the working electrode in aqueous solution containing both an electropolymerizable monomer and a biomolecule which can be physically incorporated into the growing polymer due to the immediate vicinity of the electrode surface. No additional chemicals able to promote molecule denaturation are required. Moreover, polymeric films can be easily manipulated in terms of chemical, physical and geometrical properties (*i.e.*, thickness) by the accurate selection of monomers and electrolysis charge [142]. However, this method is not applicable either when the biomolecule is sensitive to the electrostatic environment or for low amounts of components. 

Different biological materials including enzymes, antibodies, haptens, oligonucleotides and chemical receptors have been combined with EAPs [143]. Enzymes are often chosen as bioreceptors based on their specific binding capabilities as well as their catalytic activity. Many biosensors rely on immobilized enzymes that catalyze the formation of an electrochemically active byproduct from the metabolite or biomarker of interest. Conductive polymer electrodes have been frequently adopted as substrates for the enzyme coatings—layers from 10 to 200 nm thick—due to their ability to bind proteins by physical adhesion. The unique major limitation of these polymers is their hydrophobicity which can denature entrapped proteins, thus providing a sort of diffusion barrier for enzymes. However, the hydrophobic nature of some EAPs can be disguised by the incorporation of hydrophilic additives [141]. A typical application of enzyme biosensors is glucose monitoring by the use of immobilized glucose oxidase (GOx) on the electroactive polymer-coated electrode. Among different EAPs that have been employed for this aim, PPy and PEDOT are widely used, and in many cases they are doped [144,145] or nanocomposites [146,147]. The design of an efficient implantable glucose sensor for diabetes treatment able to provide continuous glucose monitoring still requires several efforts to work at physiological glucose levels. Implantable electrochemical biosensors are projected to work *in vivo*, so they mainly require that the sensing surface counteracts the deleterious adsorption of physiological biomolecules (*i.e.*, proteins), which is called biofouling [148]. Blends and co-networks of hydrogels and EAPs could be promising, stimuli-responsive and multi-functional materials to be used as substrates for *in vivo* biosensors, as widely elucidated by Guiseppi-Elie [34]. These composites consist of a hydrogel component, such as poly(hydroxymethyl metacrylate) (pHEMA) or polyethylene glycole (PEG), which, in addition to conferring the biocompatibility and anti-fouling ability [149], is able to physically or covalently immobilize biomolecules. The intimately combined EAP (mainly PPy) acts as an electroactive component to reduce interfacial electrical impedances. Oxidoreductase enzymes have been recently entrapped to conductive substrates to develop efficient amperometric biosensors. Various electrode-supported hydrogel pHEMA-PPy were synthesized to develop oxidoreductase enzyme biosensors [150]. In this case the mechanisms involve a simultaneous reaction: UV polymerization of HEMA mixed with enzymes including glucose oxidase, cholesterol oxidase or galactose oxidase, and pyrrole polymerization at the interstitial spaces of the forming hydrogel network. 

In recent years, the development of rapid and inexpensive methods for the detection of nucleotides is becoming an attractive goal to achieve because mostly DNA analysis is relevant for human health procedures, including the diagnosis of infectious diseases, genetic mutations, and drug discovery. Electrochemical techniques currently represent an optimal route to realize DNA sensors for quantitative analyses of DNA-analytes, with the formation of nucleic acid/DNA complexes on electronic transducers, *i.e.*, electrodes or semiconductors. The initial approaches have been based on redox active species used as labels for nucleotide detection [151]. An interesting example consists of a biosensor based on electroactive polyterthiophene functionalized with an oligonucleotide (ODN). A terthiophene monomer bearing a carboxyl group, 3′-carboxyl-5,2′,5′,2-terthiophene, has been recently synthesized, as a starting material for the synthesis of functionalized polyterthiophenes [152]. However, more interesting methods are represented by those where no redox indicator is necessary because changes in the DNA interface are monitored electrochemically without the use of any labels. In this context, the use of opportunely functionalized conducting polymers as substrates can allow us to achieve this type of purpose. Peng *et al.*, for example, have been proposed label-free electrochemical DNA biosensors using a novel modified PPy [153]. At this scope, an electrochemical sensor based on a co-polymer of polypyrrole functionalized with ODN was developed. In this case, poly[pyrrole-*co*-4-(3-pyrrolyl) butanoic acid] was selected as the electrode to covalently graft ODN probes by 1-ethyl-3-(3-dimethylamino) propyl carbodiimide (EDC) catalyst. It is worth enhancing the longer “linker” (butanoic acid chain) between the conducting polymer backbone and the attached ODN probe, which was introduced to act as a spacer, in order to provide lower steric hindrance to hybridization, which usually provides steric constraints in the direct adsorption on PPy films [146]. Peng has investigated the effect of the structure and saturation degree of the spacer chain on the resulting sensor properties [154], observing an increase in sensitivity of the biosensor when unsaturated and longer linkers were used in the copolymer (addressed to the extension of backbone conjugation). 

PEDOT is commonly used in biosensor applications due to its attractive properties such as high stability of the oxidized state and high electroconductivity in aqueous electrolyte solutions. An interesting study regards a new class of materials, which is molecularly imprinted polymers (MIPs), that represent a valid alternative to biological receptors because they possess an artificially created receptor structure. Indeed, a microfluidic system was developed for the detection of morphine [155] in a low range of concentrations (in the order of hundreds of micromolarity). 

PEDOT can be also polymerized with living cells to form a neural cell templated PEDOT coating with enhanced ion conductivity in “*in vitro*” culture, addressing the development of bioelectronic devices for the support of brain and heart functions [143].

In Table 2 are summarized the main properties and biomedical applications of most commonly used EAPs. 

## 4. Conclusions and Future Perspectives

The new frontier on the use of EAPs is represented by new discoveries for the design of conductive polymer tools with great potential for high throughput applications in biomedical science and medicine. The current aim is to translate between the “electron” approach of electronic devices to the “ion” approach of biology in order to design advanced systems with high bio-inspiration and automatization levels for healthcare applications. Fascinating studies have recently focused on the creation of new bioelectronic surfaces able to reduce biofilm formation by facilitating more effective bacterial killing in the case of advanced infection [156]. Innovative *in vitro* models based on organic biosensors allow detecting epithelial barrier integrity [157]. A great amount of evidence has confirmed that organic bioelectronic represents the new frontier for designing basic components of the next generation of medical devices, *i.e.*, the fabrication of high-tech brain interfaces and prostheses for a fast recovery of mobility for paralyzed patients. 

In perspective, EAPs and their use in various biomedical areas expresses an enormous potential, especially as a molecular vehicle in therapeutic/diagnostic/teragnostic applications, but the development of dedicated ion transport–based technologies to deliver biomolecules with high spatio-temporal resolution and precise electronic control is pivotal. This will produce a relevant impact in clinical surgery, by providing timely detection of disease (*i.e.*, organ regeneration, cancer therapy), moving towards the use of remote and personalized strategies based on a strict synergism between life (*i.e.*, medicine, biology) and materials (*i.e.*, chemical physics) sciences, really contributing to improving the quality of life of patients by reducing hospitalization times and current therapy invasiveness.

## Figures and Tables

**Table 1:** Structures and maximum conductivity range for some of most commonly used EAPs in doped form.

Conducting polymers		Maximum conductivity range	References
Poly(acetylene) (PAc)	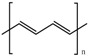	10^2^–10^3^ S∙cm^−1^	[6,11,15,39]
Poly(*p*-vinylene) (PPV)	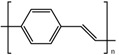		
Poly(*p*-phenylene) (PPP)	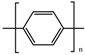	1–10^2^ S∙cm^−1^	[6,11,12,15,39]
Poly(*p*-phenylene sulphide) (PPS)	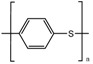		
Polypyrrole (PPy)	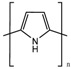		
Polyaniline (PANI)	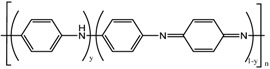		
Polythiophene (PTh)	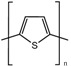		
Poly(3,4-ethylenedioxythiophene) (PEDOT)	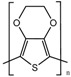	10^−1^–10 S∙cm^−1^	[12,39,40]
Poly(isothianaphtene) (PITN)	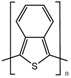		

**Table 2:** Summary of physical/chemical features of most commonly used EAPs.

EAPs	Key properties	Limitations	Main biomedical applications
PPy	High conductivity	Insolubility in organic	Neural probes
	High stability in air	solvents	Drug delivery
	Electroactivity pH [4,5,6,7,8,9,10,11]	Poor processability	Coatings
	Biocompatibility	No biodegradability	Tissue engineering
	Low solubility in water		Biosensing
			Bio-actuators
PANI	High conductivity	Low solubility in organic solvents	Drug delivery
	Enviromental stability	Electroactivity pH < 4 Poor processability	Coatings
	Suitable redox properties	No biodegradability	Tissue engineering
	Biocompatibility		Bio-actuators
PTh	High conductivity	Instability in air	Tissue engineering
	Biocompatibility	Low solubility in organic solvents	Biosensing
	High doping levels	Poor processability	Coatings
		No biodegradability	
PEDOT	High transparency	Low solubility in organic solvents	Biosensing field
	High stability in its oxidation state *vs.* biological reducing agent	Poor processability	Neural probes
	Low oxidation potential	No biodegradability	Drug delivery
	High compatibility with aqueous electrolytes		
